# Different effects of four rest periods after the last weekly training session on young male professional soccer players’ physical performance

**DOI:** 10.1371/journal.pone.0294867

**Published:** 2023-12-07

**Authors:** Mehdi Ben Brahim, Alejandro Sal-de-Rellán, Hussain Yasin, Ariadna Hernaiz-Sánchez

**Affiliations:** 1 Health and Physical Education Department, Prince Sultan University, Riyadh, Kingdom of Saudi Arabia; 2 Faculty of Health Sciences, Universidad Internacional Isabel I de Castilla, Burgos, Spain; 3 Faculty of Social Sciences and Communication, Universidad Europea de Madrid, Madrid, Spain; Instituto Politécnico de Santarém: Instituto Politecnico de Santarem, PORTUGAL

## Abstract

The main aim of this study was to analyze the optimal pre-competitive recovery period for young male professional soccer players to be in the best conditions to achieve a higher physical performance. Seventeen young male professional soccer players (age: 20.7 ± 1.0 years) who participated in the Tunisian soccer league participated in this study. Players underwent a fitness test battery after four different recovery periods: 12, 24, 36, and 48h following their last training session. The battery fitness test included a linear sprint test (i.e., 5 and 20m distances), squat jumps (SJ), countermovement jumps (CMJ), ball shooting test, a Yo-yo endurance intermittent test (YYIRT-1) and a 15-m ball dribbling agility test (Ag). The main findings indicated that the 12h recovery period resulted in better performance in the 5m sprint test compared to 36h, as well as in the SJs and CMJs as compared to the 48h recovery period. Additionally, the 24h recovery period showed significantly better results in the 20-m sprint test compared to all other recovery periods, as well as in the SJ and Ag tests compared to the 48h recovery period. In practical terms, these findings suggest that coaches should consider scheduling the last training session for soccer players 12-24h before the match to enhance their physical performance, particularly in linear sprinting, vertical jumps, and agility variables. However, in situations where it is not possible to adjust the timing of the last training session, an alternative approach is to incorporate "priming-day" exercises on the match day, around 6-8h prior to the competition.

## Introduction

Soccer is an intermittent team sport that involves a wide range of high-intensity actions such as accelerations, decelerations, changes of direction, jumping, and kicking the ball, interspersed with low-intensity periods [1,2]. Despite the limitations of the use of global positioning systems (GPS) and micro-electrical mechanical systems (MEMS) (e.g., device sampling rate, positioning and fitting of devices, satellite signal, and data-filtering methods), in the collection of reliable data [3,4], on average, a soccer player covers a total distance of 10–13 km per match, with sprints ranging from 185 to 190-m and reaching a top speed of 31 km·h^-1^ [5]. Additionally, players perform around 100–150 accelerations [6]. Given the highly physical demands of soccer, one of the primary challenges for strength and conditioning coaches is to devise effective strategies (e.g., fatigue minimization and recovery) [7,8] to enhance the physical performance of soccer players.

In the soccer training process, fatigue is constantly present due to the high demands placed on players [8]. Fatigue can be defined as the inability to maintain exercise intensity, leading to a decline in performance [9]. Previous studies have shown that physical fatigue negatively impacts soccer performance. For instance, it can result in a decrease of total distance covered and velocity [10,11], fewer accelerations and sprints [12], and reduced technical skills such as passing and shooting accuracy [13]. Therefore, technical and physical performances could be significantly impacted by fatigue [8]. To counteract its negative effects, strategies for fatigue management within soccer training routines are crucial [8]. One such strategy that has gained relevance is pre-competitive training session management [14,15]. This approach involves scheduling the final training session prior to a match on an appropriate day within the micro-cycle, facilitating recovery and enhancing the training effects [16]. However, despite the importance of this strategy, there is still no clear consensus on how to optimize its implementation [16–18]. Furthermore, regarding the scheduling of the last training session, previous studies indicated that properly designing it with a reduced pre-match interval could lead to an improvement in post-activation performance enhancement (PAPE) [16]. PAPE is a phenomenon that can improve muscle performance as a result of its contractile history following previous conditioning activity [19]. Its applicability has mainly focused on muscular power exercises (e.g., short duration, high intensity actions) [16] although it may also benefit endurance efforts (e.g., aerobic capacity) [20].

When it comes to vertical jump performance, studies have shown that its decline can persist for different durations. Some authors have observed that this may last even up to 72h after exercise [21]. However, it seems that 48h are generally sufficient for recovery in this physical variable [22,23]. Additionally, Harrison et al. [17] conducted a study and concluded that strength training involving low-volume squats at moderate load [65% of one repetition maximum (1RM)] and high load (67–87% 1RM) resulted in a 6.1% and 4.5% increase, respectively, in countermovement jump (CMJ) performance 8h later. However, it is important to note that individual characteristics may influence these responses [16]. Regarding speed and agility, research by Little and Williams [24] suggests that a recovery of 48h after the last training session is effective in minimizing fatigue in professional soccer players, as evidenced by improvements in 10-m and 20-m linear sprint times and agility test performance. This finding indicates that allowing for a 48h recovery period between intense training sessions can help reduce muscle fatigue and potentially lower the risk of injury, as highlighted by Malone et al. [25]. However, it is important to note that the optimal recovery period for aerobic capacity, agility or skills such as shooting velocity is still unknown and requires further investigation. Future studies in this area are needed to shed more light on the specific recovery strategies that would be most effective for these aspects of soccer performance.

To the authors’ knowledge, there is still no study that analyzes all physical demands together to determine the effects of four rest periods after the last weekly training session on the physical performance of male professional soccer players. Therefore, the main aim of this study was to analyze the optimal pre-competitive recovery period for young male professional soccer players to be in the best conditions to achieve a higher physical performance. Drawing from the aforementioned studies [16,18,22,26], this study’s hypothesis is that conducting the last training session 12 or 24h prior to the competition would yield the best results.

## Methods

### Participants

Seventeen young male professional male soccer players (age: 20.7 ± 1.0 years; height: 181.2 ± 4.5 cm; body mass: 74.17 ± 5.38 kg; BMI: 22.8 ± 1.5 kg/m^2^; body fat: 11.4 ± 1.8%) belonging to the same team participated in this study. This team competed in the Tunisian soccer league during the experimental period, and all players presented a minimum of 10 years of experience in competitive soccer. Players were included in the study if they completed all the assessment sessions and had not been injured for the previous 2 months. All the participants were informed of the procedures, methods, benefits, objectives, and possible risks involved in the study before giving their written informed consent. In addition, the study was performed in accordance with the Declaration of Helsinki (2013) and approved by the ethics committee of the University.

### Design and procedures

A crossover randomized design was applied to establish the best rest period between the last training session and a battery fitness test in young male professional soccer players. The intervention lasted five weeks (from 4/01/2021 to 7/02/2021). Participants completed four assessment sessions after the established recovery period in four different situations (i.e., last training session 12, 24, 36 and 48h prior to tests). Players were familiar with the battery fitness test since all of them took the warm up as a part of their daily soccer routines. To avoid interference effects, all assessment sessions were performed at the same time of day (i.e., 9:30 a.m.) and under similar environmental conditions (23–25°C), with the same sports clothes, and conducted by the same testers. Players were encouraged to maintain their nutritional routines avoiding caffeine-rich drinks such as coffee prior to assessments. This study was conducted during an in-season training camp. Data were collected by two researchers. Both researchers monitored every training session, providing verbal encouragement to each participant.

The battery fitness test was conducted in the following order: linear sprint test, squat jump test, countermovement jump test, ball shooting test, yo-yo endurance intermittent test and 15-m ball dribbling agility test. Prior to testing, players undertook a 17 min standardized warm-up, consisting of 3 min jogging, followed by 7 min of dynamic and ballistic stretching, and 7 min of progressive sprints and accelerations. During the experimental period, training weeks followed the same pattern, which is shown in Table 1.

**Table 1:** Weekly schedule during the experimental period.

Day of the week	12h daily timetable system
Monday	AM—Off
	PM—Regeneration session
Tuesday	AM—Conditioning training-Technical training
	PM—Technical and Tactical training + Game
Wednesday	AM—Technical training: Finishing (Day off Every 2 weeks)
	PM—Technical and tactical training + Game
Thursday	AM—Conditioning training—Technical training
	PM—Tactical and Technical training + Game
Friday	AM—Off
	PM—Conditioning and Technical training + Game
Saturday	AM—Off
	PM—Technical and Tactical training + Game
Sunday	PM–Assessment session

12h, last training session on Sunday AM; 24h, last training session on Saturday PM; 36h, last training session on Saturday AM; 48 h, last training session on Friday PM; *In 48h condition, Friday section is deleted*; AM, Ante Meridiem; PM, Post Meridiem.

All training sessions were completed under similar standardized conditions in a grass-soccer field where athletes usually trained and competed, while wearing their habitual garments and training shoes. Morning training sessions lasted 60 min that were performed at the same time (i.e., 9:30 a.m.), while the afternoon sessions lasted 90 min and were also performed at the same time (i.e., 17:30 p.m.). The last training session prior to assessments presented the following structure: Conditioning training (i.e., general warm-up based on jogging, static and dynamic stretching exercises; specific warm-up based on speed, shuttle running, agility, passing and dribbling drills, mobility exercises, coordination drills); Technical training (i.e., passing, drilling and shooting combination drills, agility drills); and tactical/technical training (i.e., small-sided games, tactical half-court sided games, position-specific training and real game).

### Battery fitness test

#### Linear sprint test

Participants were encouraged to complete two maximum sprints of 20-m, with a split at 5-m, allowing a 2 min of passive recovery between attempts. The players started from a standing position, 0.5-m behind the first set of photoelectric cells (Polifemo Light Radio, Microgate, Bolzano, Italy), before running at maximal speed to the second photoelectric cell. The fastest sprint was recorded and the maximum speed was calculated [27]. The intraclass correlation coefficients (ICCs) and the coefficients of variation (CVs) for the 20-m were ICC = .97, CV = 1.4%; 5m ICC = .94, CV = .7%.

#### Squat jump test and countermovement jump test

Players performed three bilateral trials of CMJs and three trials of SJs separated by 45s of passive recovery, using the My Jump 2.0 mobile application to assess jump height [28]. Every jump was recorded at 240 Hz with an iPhone 8 Plus mobile device (Apple Inc, Cupertino, CA, USA). During the CMJs, players were instructed to perform a downward movement followed by a complete, explosive extension of the lower limbs, maintaining arms akimbo. However, for SJs, players must go down, wait, and then, perform an explosive extension of the lower limbs. For the subsequent analysis, the highest jump for each test was selected. Reliability values were CMJ ICC = .97, CV = 3.3%; SJ ICC = .98, CV = 2.8%.

#### Ball shooting test

Players were encouraged to perform a maximal velocity instep kick of a stopped ball toward a 1 x 1-m target [29]. Players were allowed five attempts to strike the ball as hard as possible with a 1-minute rest between each attempt. Ball speed was measured by a radar gun located 3-m away from the stopped ball and pointing toward the target according to the instructions manual (Sports Radar Gun SRA 3000; Precision Training Instrument, IL). Participants wore their own soccer shoes and a statutory ball was used (Adidas, Germany; 69.0 ± 0.2 cm in circumference and 440 ± 0.2 g in mass). The highest ball speed recorded was selected for the subsequent analysis. Reliability values for Ball shooting test were ICC = .84, CV = 1.6%.

#### Yo-yo endurance intermittent test

The YYIR1 consisted of 2 x 20-m runs back and forth between two lines at a progressively increasing speed controlled by audio bleeps [26]. When the players failed twice to reach the corresponding line in time, the distance covered was recorded and represented the test result. Each bout was interspersed with a 10 s active rest period consisting of 2 x 5-m jogging. The total distance covered during the YYIR1, including the last incomplete shuttle, was taken as the testing scores. Reliability values for YYIR1 were ICC = .87, CV = 3%.

#### 15-m ball dribbling agility test

This test was performed according to the protocol previously described by Mujika et al. [30] using photoelectric cells (Polifemo Light Radio, Microgate, Bolzano, Italy) to register the time needed to cover it. Each player performed two maximal repetitions interspersed with 3 min of passive recovery. Players were required to drive a ball while performing the test. After the slalom section, the ball was kicked under the hurdle while the player cleared it. The player then freely kicked the ball towards either of two small goals placed diagonally 7-m on the left and the right sides of the hurdle and sprinted to the finish line. The fastest repetition was selected for further analysis. Reliability values for 15-m ball dribbling agility test were ICC = .74, CV = 2.6%.

#### Statistical analysis

Descriptive data are presented as mean ± standard deviations (SD). The Kolmogorov-Smirnov and Levene tests were conducted to prove the normality of data distribution and the homogeneity of variances. All data were normally distributed. A repeated measure ANOVA was applied to detect differences in the battery fitness test regarding each recovery period (i.e., last training session 12, 24, 36 and 48h prior to tests). Eta-squared (η^2^) effect sizes for group interaction were calculated. An effect of η^2^ ≥ 0.01 indicates TM as small, ≥ 0.059 as medium, and ≥ 0.138 as large effect, respectively [31]. When significant differences were observed, a post hoc’s with Bonferroni corrections was applied. The magnitude of differences was calculated using Hedges’ g between different devices. The g values were interpreted as trivial (g < 0.2), small (g < 0.5), moderate (g < 0.8) and large (g ≥ 0.8) [31]. Data were analyzed using the Statistical Package for Social Sciences (SPSS 27.0, SPSS Inc., Chicago, IL, USA), and the statistical significance was set at *p* < 0.05.

## Results

The differences in the battery fitness test after each recovery period are presented in Table 2. The ANOVA (F = 4191.56, *p* = 0.001, η^2^ = 0.89) shows significant differences in favor of 12h recovery period in SP5 as compared to 36h (F_3,48_ = 4.079, *p* = 0.009, ES = -0.697, moderate) and in SP20 as compared to 24h (F_3,48_ = 10.958, *p* < 0.001, ES = 0.216, small), and in SJ (F_3,48_ = 4.953, *p* = 0.004, ES = 0.251, small) and CMJ (F_3,48_ = 4.205, *p* = 0.010, ES = 0.214, small) as compared to 48h. Regarding the 24h recovery period, significant better results were obtained in SP20 as compared to 36h (F_3,48_ = 10.958, *p* < 0.001, ES = - 0.312, small) and 48h (F_3,48_ = 10.958, *p* < 0.001, ES = - 0.385, small), and in SJ (F_3,48_ = 4.953, *p* < 0.004, ES = 0.199, trivial) and Ag (F_3,48_ = 3.711, *p* < 0.018, ES = - 0.158, trivial) as compared to 48h. Finally, no statistically significant differences were observed in Ball Vel and YYIRT-1 in any recovery period. Mauchly’s Sphericity test indicates that the assumption of sphericity is not met (W = 1.565, *p* = 0.001). The homoscedasticity analysis revealed no significant deviations.

**Table 2:** Descriptive data (mean ± SD) and differences between recovery periods.

Variable	12h	24h	36h	48h
SP5 (s)	1.07 ± 0.04^b^	1.08 ± 0.07	1.11 ± 0.09	1.09 ± 0.06
SP20 (s)	4.04 ± 0.18^a^	3.99 ± 0.20^bc^	4.05 ± 0.17	4.08 ± 0.20
SJ (cm)	31.73 ± 4.64^c^	31.49 ± 4.83^c^	31.19 ± 4.58	30.58 ± 4.29
CMJ (cm)	33.45 ± 5.01^c^	32.94 ± 4.70	32.94 ± 4.34	32.47 ± 4.11
Ball vel (km·h^-1^)	83.18 ± 6.61	82.82 ± 7.59	80.65 ± 4.20	79.45 ± 6.13
YYIRT-1 (m)	2689.41 ± 330.46	2683.53 ± 339.43	2681.18 ± 319.18	2678.82 ± 361.14
Ag (s)	3.95 ± 0.29	3.93 ± 0.32^c^	3.96 ± 0.31	3.98 ± 0.33

SD, standard deviation; 12h, last training session performed 12 hours before assessment; 24h, last training session performed 24 hours before assessment; 36h, last training session performed 36 hours before assessment; 48h, last training session performed 48 hours before assessment; SP5, 5m linear sprint test; SP20, 20m linear sprint test; SJ, squat jump; CMJ, countermovement jump; Ball vel, ball shooting test; YYIRT-1, Yo-yo endurance intermittent test; Ag, 15-m ball dribbling agility test.

^a^ significant differences with 24h (*p* < 0.05); ^b^ significant differences with 36h (*p* < 0.05); ^c^ significant differences with 48h (*p* < 0.05).

## Discussion

This study aimed to analyze the optimal pre-competitive recovery period for young male professional soccer players to be in the best conditions to achieve a higher physical performance. It is the first study to comprehensively analyze the effects of four different rest periods following the last weekly training session in young male professional soccer players. The main findings favored the 12h recovery period in SP5 compared to 36h, as well as in SJ and CMJ compared to 48h. Additionally, the 24h recovery period yielded significantly better results in SP20 as compared to all other recovery periods, as well as in SJs and Ag compared to 48h.

Regarding the linear sprint performance, this study’s findings demonstrated superior results in SP5 when a 12h recovery period was implemented compared to the 36h recovery period. This supports the idea that a well-designed last training session with a reduced gap before a match could lead to PAPE, mainly in short duration and high-intensity actions [16]. On the other hand, significant differences favoring the 24h recovery period were observed compared to all other conditions. For longer sprint distances (i.e., 20 m), a 12h recovery period may be insufficient as it may not allow for complete regeneration following induced fatigue [32]. Moreover, recovery periods of 36 and 48h appear to be excessively long, potentially diluting the physiological mechanisms activated through training (e.g., increased fiber sensitivity to calcium ions or activation of high-frequency motor neurons), thereby impairing the PAPE effect. Therefore, it is advisable to select shorter recovery periods (i.e., 12 or 24h) to elicit a positive delayed effect on sprint performance in professional soccer players.

The present findings suggest that 12h and 24h recovery periods are sufficient to achieve optimal performance in vertical jumps compared to a 48h recovery period. These results are similar to those of others studies [33,34]. For soccer players specifically, it was concluded that the application of “priming-training” 24h before a competition could improve vertical jump performance (i.e., SJ and CMJ) [34]. Similarly, positive effects on vertical jump performance were also found in young swimmers after 24h of rest [33]. This finding could support the idea of PAPE effects in vertical jumps after a reduced recovery period for male professional soccer players [35].

Regarding agility, the authors’ results indicate that the best performance in the agility test was observed after a 24h recovery period. This upholds the notion that this recovery period optimizes the PAPE effects while minimizing the impact of fatigue. It is worth highlighting that the shooting ability is associated with successful professional soccer players, as some authors indicated [36]; thus, its optimization is justified. In this study it was observed that although the results were not statistically significant compared to the other recovery periods, the best ball speed performance was obtained at 12h while performance decreases when the recovery period is increased. This result confirms the significant PAPE effect of a reduced recovery period on short-duration and high-intensity actions. Finally, no differences in aerobic capacity were observed, noting that PAPE has little influence on this capacity. These results point at the need to analyze in future studies the optimal parameters to maximize benefit from PAPE, and the role it can play in optimal performance while interacting with central and peripheral factors associated with muscle fatigue. In this sense, further studies should elucidate the association between PAPE responses and adaptations on aerobic capacity for professional soccer players [37].

This study has several limitations that practitioners should be aware of. Firstly, physical performance was assessed using fitness tests rather than during actual matches. While fitness tests provide valuable information, it is essential to complement these findings with studies that assess performance in real match conditions (i.e., analyzing the external load). Another limitation is the lack of consideration for individual fitness levels, which could potentially influence the results obtained. Additionally, the analysis did not consider the playing position of each participant, although it is well-known that the physical demands vary among different positions in soccer [38]. Moreover, important factors such as sleep quality and quantity, which have a significant impact on recovery and fatigue after training [7,39], were not assessed in this study, therefore it should be monitored in future research. Finally, other variables (e.g., hematological, biochemical or nutritional) should be collected and analyzed in future research too, as they play an important role in the recovery of professional soccer players [40].

## Conclusions

The present findings indicate that the 12h recovery period yielded superior results in SP5 as compared to the 36h recovery period, while both SJs and CMJs exhibited better performance outcomes after the 12h recovery period when compared to the 48h recovery period. Additionally, in the case of the 24h recovery period, significantly improved results were observed in the SP20 variable compared to all the other recovery periods, and both the SJ and Ag variables showed better performance outcomes as compared to the 48h recovery period. These results suggest that a shorter recovery period (12h) may be sufficient for optimizing performance in certain variables, such as SP5, SJ, and CMJ, as compared to longer recovery periods. Furthermore, a 24h recovery period seems to be particularly advantageous, as indicated by superior performance in SP20, SJ, and Ag compared to other recovery periods.

In practical terms, the results in this study suggest that coaches and strength and conditioning professionals should consider scheduling the last training session for soccer players 12-24h before the match to enhance their physical performance, particularly in linear sprinting, vertical jumps, and agility variables. However, in situations where it is not possible to adjust the timing of the last training session, an alternative approach is to incorporate "priming-day" exercises (e.g., high-intensity resistance exercises through high loading (≥ 85% 1 repetition maximum) or ballistic exercises at lower loads) on the match day, around 6-8h prior to the competition [41]. These exercises are designed to induce a PAPE effect, which can lead to improved short-term performance in high-intensity actions.

## Supporting information

S1 File (CSV)
